# Expression of Oxidative Phosphorylation Complexes and Mitochondrial Mass in Pediatric and Adult Inflammatory Bowel Disease

**DOI:** 10.1155/2022/9151169

**Published:** 2022-01-06

**Authors:** Anna M. Schneider, Mihriban Özsoy, Franz A. Zimmermann, Susanne M. Brunner, René G. Feichtinger, Johannes A. Mayr, Barbara Kofler, Daniel Neureiter, Eckhard Klieser, Elmar Aigner, Sebastian Schütz, Nathalie Stummer, Wolfgang Sperl, Daniel Weghuber

**Affiliations:** ^1^Department of Pediatrics, University Hospital Salzburg, Paracelsus Medical University, Salzburg, Austria; ^2^Department of Pathology, University Hospital Salzburg, Paracelsus Medical University, Salzburg, Austria; ^3^First Department of Medicine, University Hospital Salzburg, Paracelsus Medical University, Salzburg, Austria; ^4^Department of Mathematics, Paris Lodron University, Salzburg, Austria

## Abstract

**Introduction:**

Inflammatory bowel disease (IBD), which includes Crohn's disease (CD) and ulcerative colitis (UC), is a multifactorial intestinal disorder but its precise etiology remains elusive. As the cells of the intestinal mucosa have high energy demands, mitochondria may play a role in IBD pathogenesis. The present study is aimed at evaluating the expression levels of mitochondrial oxidative phosphorylation (OXPHOS) complexes in IBD. *Material and Methods*. 286 intestinal biopsy samples from the terminal ileum, ascending colon, and rectum from 124 probands (34 CD, 33 UC, and 57 controls) were stained immunohistochemically for all five OXPHOS complexes and the voltage-dependent anion-selective channel 1 protein (VDAC1 or porin). Expression levels were compared in multivariate models including disease stage (CD and UC compared to controls) and age (pediatric/adult).

**Results:**

Analysis of the terminal ileum of CD patients revealed a significant reduction of complex II compared to controls, and a trend to lower levels was evident for VDAC1 and the other OXPHOS complexes except complex III. A similar pattern was found in the rectum of UC patients: VDAC1, complex I, complex II, and complex IV were all significantly reduced, and complex III and V showed a trend to lower levels. Reductions were more prominent in older patients compared to pediatric patients and more marked in UC than CD.

**Conclusion:**

A reduced mitochondrial mass is present in UC and CD compared to controls. This is potentially a result of alterations of mitochondrial biogenesis or mitophagy. Reductions were more pronounced in older patients compared to pediatric patients, and more prominent in UC than CD. Complex I and II are more severely compromised than the other OXPHOS complexes. This has potential therapeutic implications, since treatments boosting biogenesis or influencing mitophagy could be beneficial for IBD treatment. Additionally, substances specifically stimulating complex I activity should be tested in IBD treatment.

## 1. Introduction

Inflammatory bowel disease (IBD) with its two major clinical forms, ulcerative colitis (UC) and Crohn's disease (CD), is a chronic relapsing inflammatory disorder of the intestine [1]. UC is defined as a continuous and superficial, mucosal, and submucosal inflammation limited to the colon. CD, on the other hand, is characterized by scattered lesions affecting any part of the gastrointestinal tract, with transmural inflammation associated with many complications [2]. Although the exact etiology of IBD is still enigmatic, it is known to be a multifactorial disease that results from a complex interplay of genetic susceptibility, an altered immune response, changes in the intestinal microbiota, and environmental triggers [3]. Destruction of the intestinal epithelial barrier, increased permeability, dysfunctional immunoregulation, and increased invasion by immune cells are central disease mechanisms [4–6]. Although IBD may develop at any age, up to 25% of patients are diagnosed during childhood or adolescence [7, 8]. Previous reports have suggested that the age of disease onset correlates inversely with disease outcome, indicating that younger age may be an important risk factor for aggressive, treatment-resistant disease [9].

Mitochondria supply cells with energy by producing adenosine triphosphate (ATP) via the action of oxidative phosphorylation (OXPHOS) complexes I to V [10]. Cells with high energy demand, like intestinal epithelial cells, tend to be more vulnerable to the consequences of mitochondrial dysfunction. Several possible links between mitochondria and IBD have been reported. For example, elevated levels of reactive oxygen species (ROS) were observed in mitochondria of patients with IBD [11, 12], highlighting oxidative injury in IBD pathology. Furthermore, recent data suggest that mitochondrial dysfunction compromises the intestinal barrier and may contribute to IBD pathogenesis [11, 13–18]. Additionally, IBD patients have significantly higher levels of circulating mitochondrial DNA (mtDNA) in their plasma and feces. Moreover, their mtDNA levels correlate with the severity and activity of the disease, and mtDNA was found to serve as a proinflammatory damage-associated molecular pattern during active IBD [19]. Regarding mitochondrial function and morphology in the gut, several studies showed a significant deficiency in the enzyme activity of OXPHOS complexes as well as changes in morphological appearance of mitochondria [17, 18]. Another study reported loss of cytochrome c oxidase (COX; complex IV) to be an indicator of tumor progression in adults with UC [20]. Studies of mitochondrial dysfunction in pediatric IBD are scarce: there are two clinical cases and one study that focused on the interplay between the intestinal microbiota and mitochondria [21–23]. Specific mitochondrial proteins were shown to be the primary proteins downregulated in IBD, suggesting a regulatory interaction between mitochondria and the intestinal microbiota as well as a resulting imbalance of this relationship in CD patients. More recently, transcriptome analysis of rectal mucosal specimens revealed downregulation of mitochondria related genes in a large pediatric UC cohort [24].

**Figure 1:**
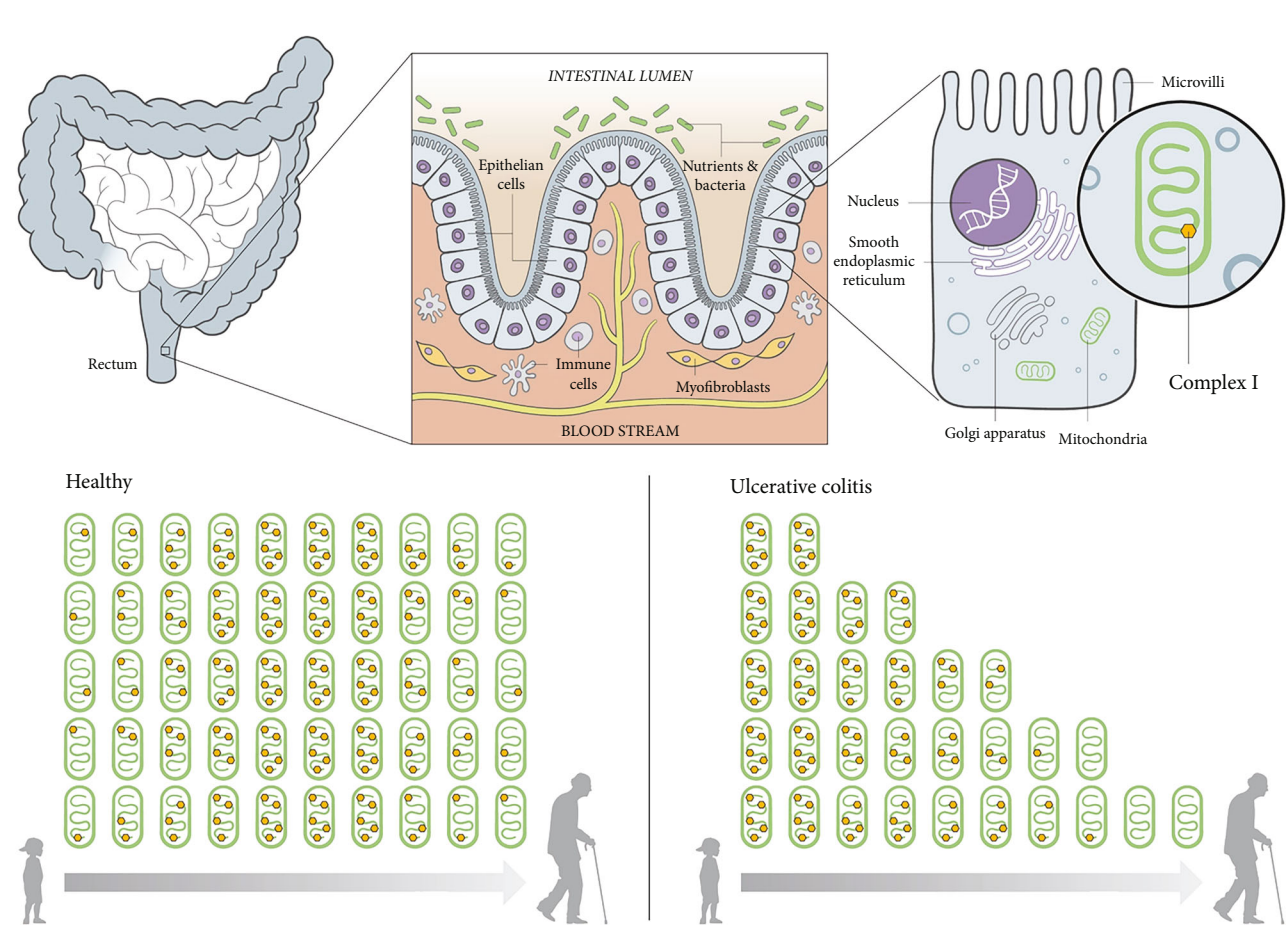
Overview and results of complex I in ulcerative colitis. The rectum, which is one of the main affected sites in ulcerative colitis, is located in the last part of the large intestine (a). The luminal surface of the intestine is formed by an epithelial layer, which is composed of simple columnar epithelial cells separating the intestinal lumen from the blood stream. It is responsible for the absorption of useful substances and acts as a barrier in preventing harmful substances migrating from the gut to other parts of the body (b). The epithelium is composed of simple columnar epithelial cells, which contain numerous mitochondria and other organelles. The mitochondria are composed of inner and outer membranes. Proteins in the latter, like VDAC1, represent the mitochondrial mass. The inner membrane harbors the respiratory chain with its complexes and is responsible for producing energy via oxidative phosphorylation (c). In a healthy state, mitochondrial mass stays stable across the life span and the average protein expression levels of the respiratory chain subunits, here represented by complex I, increase continuously from childhood onward, peak in middle age, and decline thereafter (d). In ulcerative colitis, a trend of continuous deterioration of mitochondrial mass and complex I is seen from childhood onward (e).

The aim of this study was to investigate if there are defects in OXPHOS complex expression in IBD, whether the grade of inflammation correlates with alterations in OXPHOS expression, and whether there are differences in OXPHOS expression in pediatric versus adult IBD (Figure 1).

## 2. Material and Methods

The study was approved by the local ethics committee *(415-E/2080/5-2016)* and conducted in accordance with the Helsinki Declaration of 1975 (revised 2013).

### 2.1. Patients and Tissue Samples

Patients with chronic abdominal pain, elevated fecal calprotectin, or signs and symptoms of IBD underwent diagnostic colonoscopy. In routine ileocolonoscopies performed at the hospital, biopsy specimens were taken from the intestinal epithelium in a stepwise approach starting from the terminal ileum and including all segments of the colon (the cecum, ascending colon, transverse colon, descending colon, sigmoid colon, and rectum). The specimens were then analyzed by pathologists and used by clinicians to confirm the diagnosis. The remaining biopsy material was obtained from the Institute of Pathology, University Hospital Salzburg, Austria, and used for the present study.

If the diagnosis of IBD was established by macroscopic and histologic evaluation, patients were assigned to our study group. If colonoscopy did not show any abnormalities on gross or on microscopic analysis, patients were assigned to the control group. A pathologist verified that the samples in the control group had no abnormalities by using a histologic severity score (HSS) from 0 to 3, which included evaluation of crypt architecture (0-3), acute and chronic inflammation (each 0-3), and regeneration of the epithelium (0-3), resulting in a range of integer values from 0 to 12 before the average of all 4 components was determined. Only samples with an average score of less than or equal 1 were included in the control group. Some samples of the control group were used previously [25].

As CD mainly affects the terminal ileum, one biopsy per CD patient was obtained from that location. Similarly, the rectum is the most commonly affected site in UC, so one rectal biopsy per patient was obtained in the UC cohort. From each patient, a biopsy from the ascending colon was used as well, to enable comparison of all study groups based on the same anatomical site. Samples from the terminal ileum, ascending colon, and rectum were examined in the control group.

In the diseased groups (pediatric and adult IBD), only newly diagnosed patients were included, ensuring that these patients did not receive any prior IBD-specific treatment, which may have influenced the results.

### 2.2. Immunohistochemistry

The samples were fixed in 4% neutral- buffered formalin and embedded in paraffin and cut in 4 *μ*m sections with a microtome, and three samples each were placed on a specimen holder. Immunohistochemical (IHC) staining was performed as previously described by Zimmermann et al. [26, 27]. The following antibodies were used: mouse monoclonal anti-complex I subunit NDUFS4 (WH0004724M1-100UG, dilution 1 : 1000; Sigma Aldrich, St. Louis, USA), mouse monoclonal anti-complex II subunit SDHA (ab14715, 1 : 3000; Abcam, Cambridge, UK), mouse monoclonal anti-complex III subunit core 2 (UQCRC2; ab14745, 1 : 2000; Abcam, Cambridge, UK), mouse monoclonal anti-complex IV subunit I (MT-CO1; ab14705, 1 : 1000; Abcam, Cambridge, UK), mouse monoclonal anti-complex V subunit alpha (ATP5F1A; ab14748, 1 : 2000; Abcam, Cambridge, UK), and mouse monoclonal porin antibody subunit VDAC1 (ab14734, 1 : 2000; Abcam, Cambridge, UK). All primary antibodies were diluted in Dako antibody diluent with background-reducing components (Dako, Glostrup, Denmark). Digital micrographs were taken with a Moticam 5+ camera using Motic Images Plus 2.0 software (Motic, Wetzlar, Germany).

### 2.3. Evaluation and Statistics

**Figure 2:**
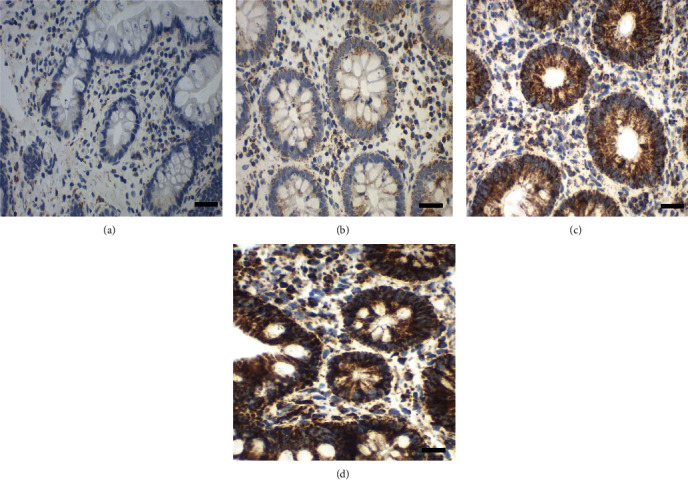
Assessment of staining intensities (0 to 3) with complex I antibody (subunit NDUFS4). Staining intensity 0: no staining, due to no primary antibody control (a, case number 7229); 1: weak staining (b, case number 41197); 2: moderate staining (c, case number 33837); and 3: strong staining (d, case number 5905). Scale bar 100 *μ*m.

The staining intensity and overall protein expression (percentage of cells staining positive) of OXPHOS complexes I-V and VDAC1 (porin) for each sample were assessed by two independent examiners blinded to each other and to information regarding the diagnosis or patient's age. A scoring system (0: no staining; 1: weak staining; 2: moderate staining; and 3: strong staining) was used to quantify the levels of staining intensity (Figure 2). The intensities were multiplied by the percentage of positive cells present in the specimen to yield score values, as semiquantitative indicators for expression level [27].

### 2.4. Statistical Analysis

Epidemiological data were analyzed descriptively. Due to the ordinal data level, expression scores were compared by median and interquartile range (IQR), and graphical representation was done by boxplots and scatterplots. Expression levels were compared in multivariate models per intestinal section by the nonparametric method of Dobler et al., including disease (CD, UC) vs. controls, age (pediatric vs. adults), and the interaction in all models [28]. *p* values of these three models were corrected using the Bonferroni method.

In a next step, expression levels were analyzed separately for each complex to examine complex-specific differences [29]. This was done first for all IBD patients vs. controls (CD in the terminal ileum, UC in the rectum and CD and UC in the ascending colon). In a second step, samples were stratified by age. As healthy controls have already been compared through their lifespan [25], only IBD patients were included in this study. Nonparametric ANOVA-type statistics were used to examine variable effects. Due to the exploratory style of this further analysis, no *p* value correction was applied. For the ascending colon, post hoc analysis was done when results were significant so as to distinguish exactly between the three groups.

In a final step, expression levels were stratified by degree of inflammation level (HSS), and correlation analysis for age and stage was done. By definition, the control group had HSS levels less than 1, whereas CD and UC patients had scores from 1 to 3.

All tests were carried out at the 5% significance level. All calculations were performed using R software (version, 4.0.2).

## 3. Results

### 3.1. Patient Characteristics

In total, 286 samples from 124 probands (34 CD, 33 UC, and 57 controls) were stained immunohistochemically for all five OXPHOS complexes and VDAC1 (Table 1). 19 samples had to be excluded due to low quality of the sample or no crypts on the sample. The mean age was 38 years, and 46 patients were below 18 years of age, 58% being female and 42% male. The patients were divided into 4 subgroups: pediatric patients with IBD (p-IBD), adult patients with IBD (a-IBD), children and adolescents without IBD or any inflammation (pediatric controls), and adults without IBD or any inflammation (adult controls).

Regarding evaluation, the average interobserver variability between the two examiners was 0.24 points within the intensity of the staining and 6.31% regarding the percentage of positive cells.

For further statistical analysis, multivariate models per intestinal segment examined all complexes and VDAC1 simultaneously, with group (CD, UC, and controls) and age (pediatric and adult) as covariates first.

### 3.2. Crohn's Disease (Terminal Ileum)

**Figure 3:**
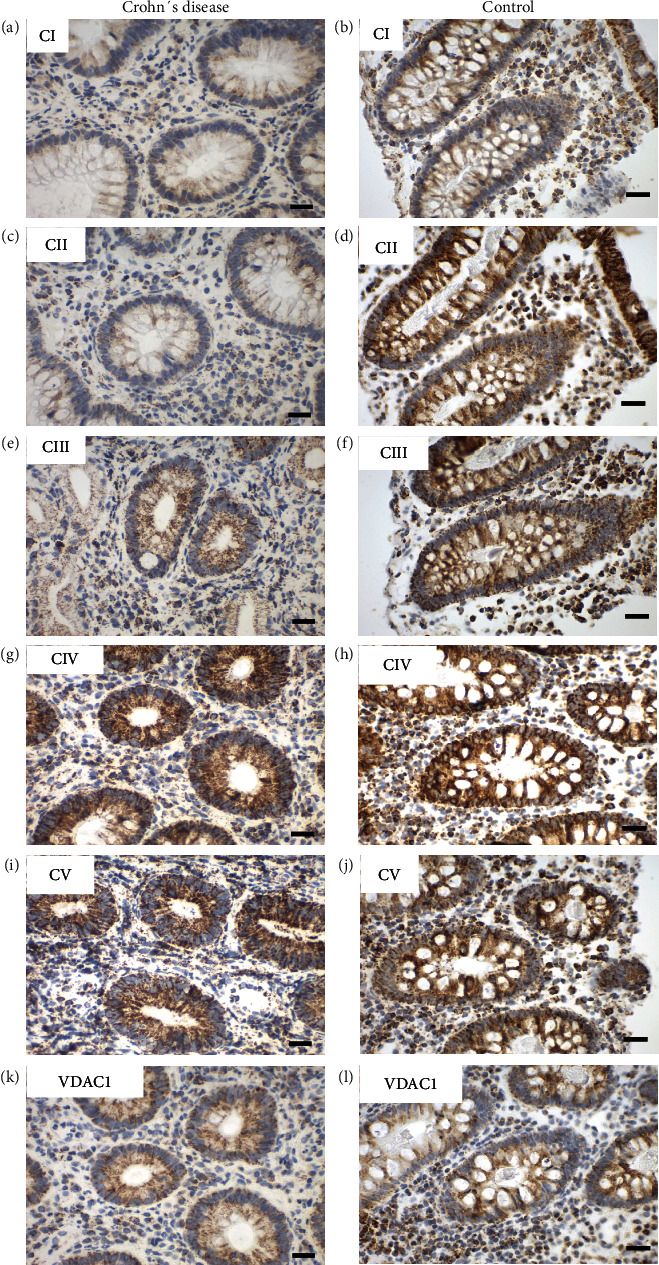
Representative images of immunohistochemical staining of complex I-V and VDAC 1 in the terminal ileum in pediatric Crohn's disease (CD) (case number 11760) and a control (case number 18642). (a, b) Complex I, (c, d) complex II, (e, f) complex III, (g, h) complex IV, (i, j) complex V, and (k, l) VDAC1 (porin). Weaker staining is present in complex I and II and VDAC1 in CD. Scale bar 100 *μ*m.

To elucidate whether there are differences in the expression of subunits of the OXPHOS complexes I to V and VDAC1 samples of the terminal ileum of CD patients and age-matched controls were investigated (Figure 3).

VDAC1 levels were moderately diminished by 7% compared to controls. Consistently, levels of subunits of the OXPHOS complexes were diminished by 10-26% with exception of complex III subunit UQCRC2 in CD (Table 2). Across all age groups, CD patients have a reduction of almost 30% in complex II levels compared to that of age-matched controls. The magnitude of the reduction of the NDUFS4 subunit of complex I was equal (21% reduction). However, it was not significant because of a higher standard deviation.

**Figure 4:**
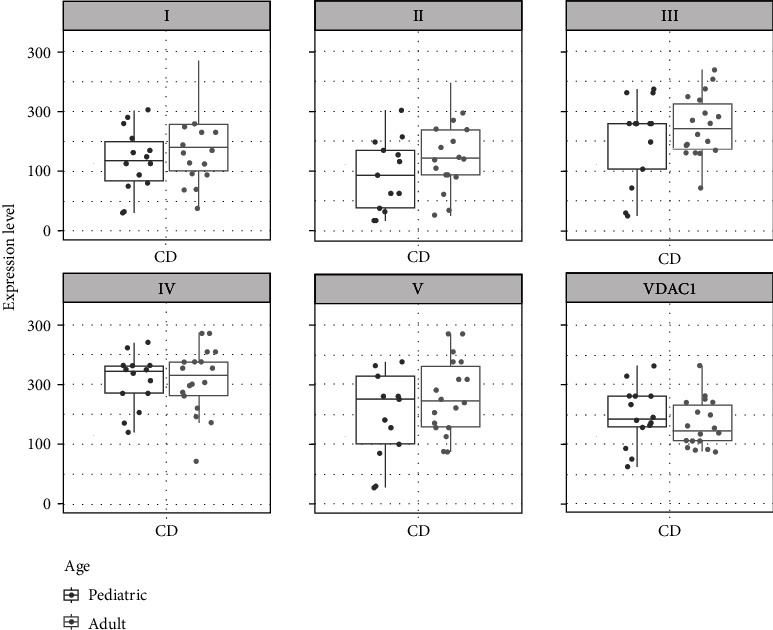
Boxplots and scatterplots showing expression levels of OXPHOS complexes I-V and VDAC1 (porin) in the terminal ileum in Crohn's disease (CD) in pediatric and adult patients.

When CD patients were stratified by age, no significant differences in the expression levels of all complexes were seen between pediatric and adult patients. However, minor trends to higher levels were present for all subunits of the OXPHOS complexes. In contrast mitochondrial mass (VDAC1) tended to be lower with increasing age (Figure 4).

**Figure 5:**
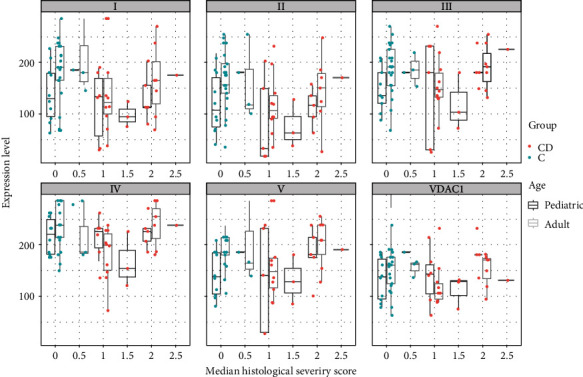
Boxplots and scatterplots of expression levels of OXPHOS complexes I-V and VDAC1 (porin) stratified by age and histological severity score (HSS) in the terminal ileum in Crohn's disease (CD) and controls (C). 0 indicates no histological abnormalities, 2.5 severe signs of inflammation.

To evaluate a potential connection between mitochondrial expression and inflammation, score values of expression levels were stratified by degree of inflammation (HSS). The control group showed high mitochondrial expression levels, comparable to those of CD patients with higher inflammation, while the lowest expression levels were found in the moderate inflammation group of CD patients (HSS 1.5). Adults tended to have a higher correlation of inflammation and mitochondrial expression level, while children showed a weaker correlation (spearman correlation 0.312 vs. 0.154; Figure 5).

### 3.3. Ulcerative Colitis (Rectum)

**Figure 6:**
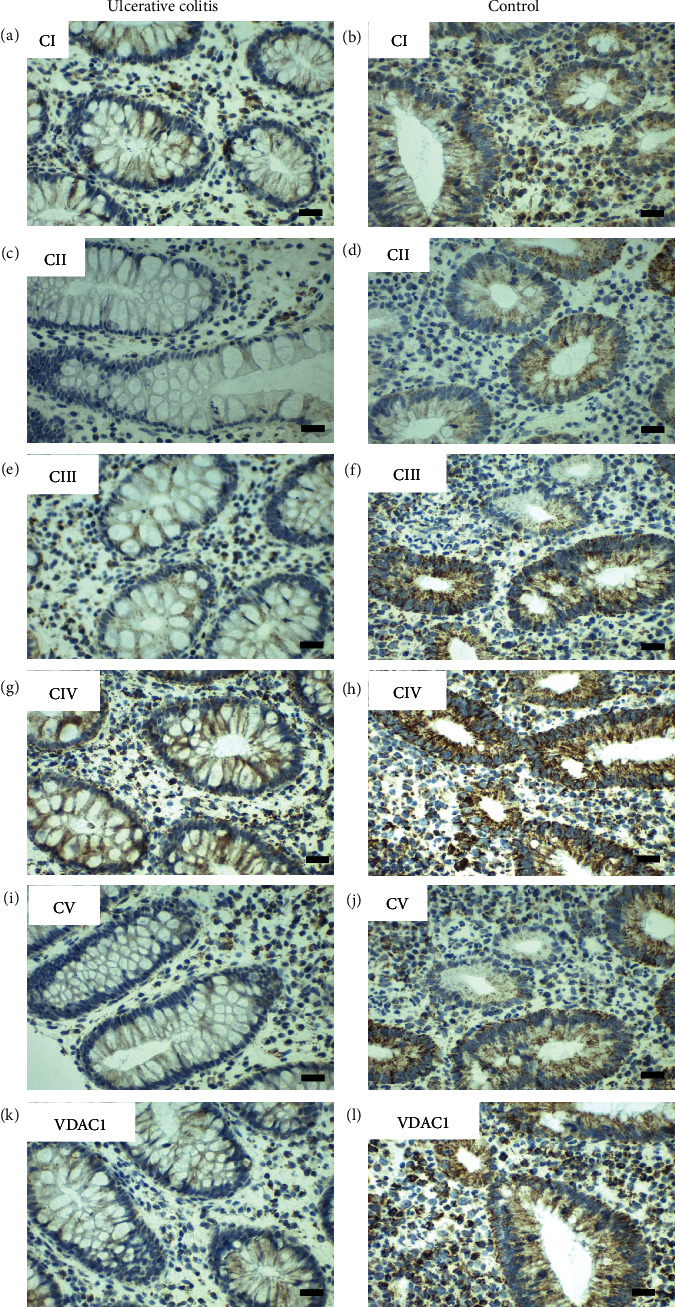
Representative images of immunohistochemical staining of complex I-V and VDAC 1 in the rectum in pediatric ulcerative colitis (UC) (case number 18385) and a control (case number 1867). (a, b) Complex I, (c, d) complex II, (e, f) complex III, (g, h) complex IV, (i, j) complex V, and (k, l) VDAC 1 (porin). Weaker staining is seen in all complexes of the UC patient. Scale bar 100 *μ*m.

IHC staining of subunits of the complexes I to V and VDAC1 in the rectum was done in tissue samples from UC patients and age-matched controls (Figure 6). VDAC1 was significantly reduced in UC patients compared to controls (*p* = 0.009; reduction by 24%). Consistently, all chosen subunits of the OXPHOS complexes were diminished by 12-43% compared to controls (Table 3). Subunits of complex I, complex II, and complex IV were significantly lower, whereas complex III and V only showed a trend to lower levels.

**Figure 7:**
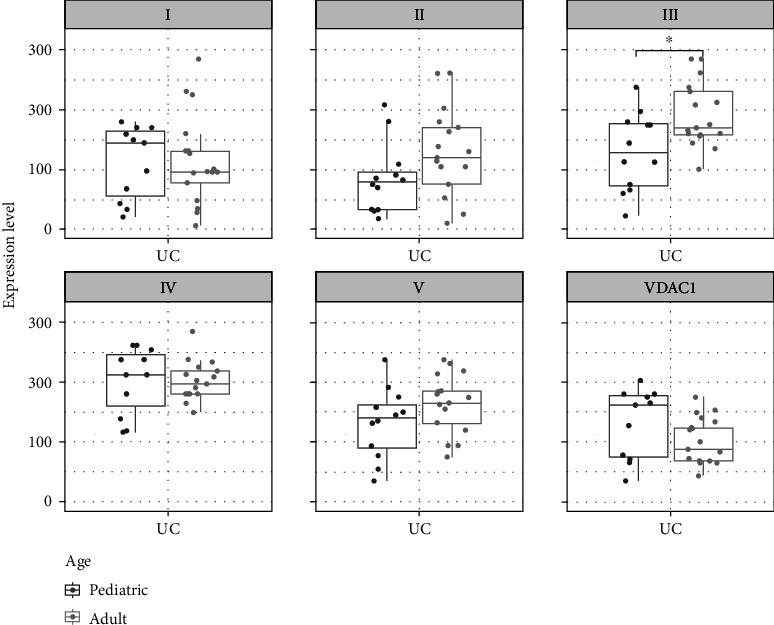
Boxplots and scatterplots showing expression levels of OXPHOS complexes I-V and VDAC1 (porin) in the rectum of pediatric and adult ulcerative colitis (UC) patients. ^∗^Significance (*p* < 0.05).

Stratification of UC patients by age revealed a trend to lower expression for complexes I and IV and VDAC1 in adults, whereas higher expression with increasing age was seen for complex II, III, and V (Figure 7).

**Figure 8:**
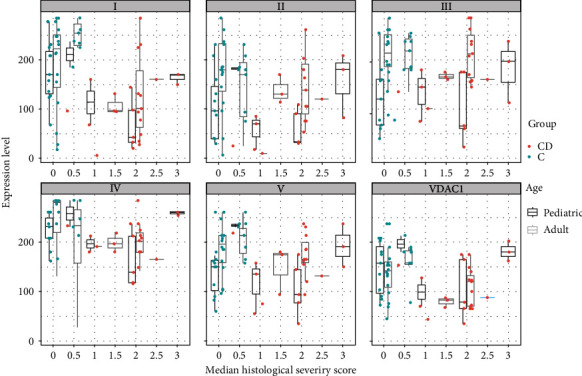
Boxplots and scatterplots of expression levels of OXPHOS complexes I-V and VDAC1 (porin) stratified by age and histological severity score (HSS) in the rectum in ulcerative colitis (UC) patients and controls (C). 0 indicates no histological abnormalities, 3 severe signs of inflammation.

Score values of expression levels stratified by degree of inflammation showed an undulating course of expression levels for different degrees of inflammation, but like in CD, patients with the highest HSS inflammatory scores exhibited higher mitochondrial expression levels compared to patients with only moderate degrees of inflammation. Children showed a trend for higher correlation of inflammation and expression level, whereas adults tended to have a weak correlation (Spearman correlation 0.419 vs. 0.165, Figure 8).

### 3.4. Comparison of IBD Patients and Controls in the Ascending Colon

In the ascending colon, all IBD patients (CD and UC) exhibited lower levels of complexes I, II, and V and VDAC1 compared to age-matched controls. Complex II was most affected, showing a reduction of 51%, followed by complex I, which was reduced by 36%, VDAC1 reduced by 34%, and complex V reduced by 23% compared to the expression levels of the control group. No difference was identified between the groups in the expression of complexes IV and V (Table 4).

**Figure 9:**
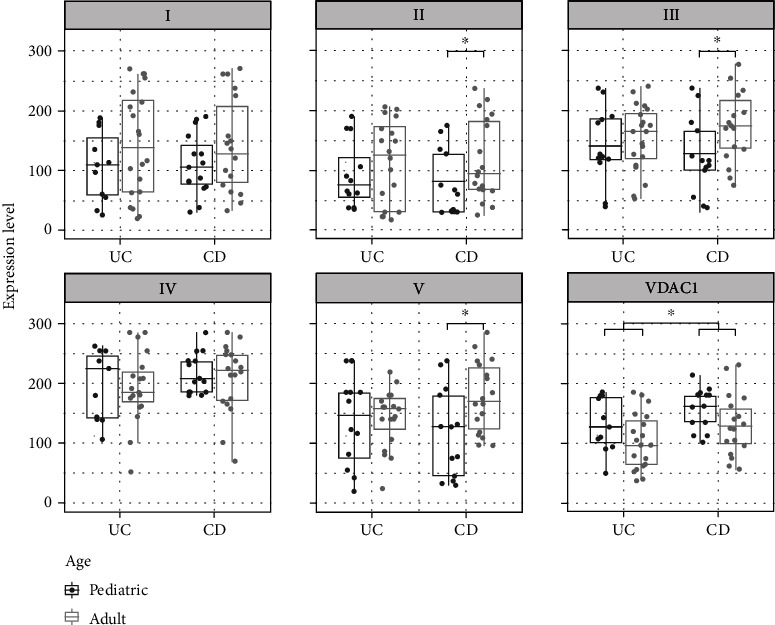
Boxplots and scatterplots showing expression levels of OXPHOS complexes I-V and VDAC1 (porin) in the ascending colon in Crohn's disease (CD) and ulcerative colitis (UC) pediatric and adult patients. ^∗^Significance (*p* < 0.05).

A reduction in VDAC1 was found for pediatric IBD patients versus adult IBD patients (*p* < 0.05). Stratified by disease subtype, adult CD patients showed higher expression levels of complexes II, III, and V, and a trend in complex I and IV, but lower expression of VDAC1 compared to pediatric patients. In the UC-subgroup, expression in adults tended to be lower for complex IV and VDAC1 and higher for all other complexes, but no significant differences were found (Figure 9).

## 4. Discussion

The present study characterized OXPHOS expression and mitochondrial mass in the intestinal mucosa of children and adults with IBD compared to controls. The expression levels of OXPHOS complexes in healthy individuals were recently shown to increase from childhood onward and then decline in older subjects [25]. These data suggest that reductions in the levels of mitochondrial OXPHOS complexes in intestinal crypts might be transiently compensated in adulthood, but that, ultimately, reduced expression occurs in persons aged 60 years and older.

In IBD, the levels of OXPHOS subunits were reduced across all age groups. The defects were more frequent in adult than in pediatric patients and more prominent in UC than in CD (Figure 1). A significant reduction of VDAC1 was present in UC compared to controls, and there was a trend to lower levels in CD. VDAC1 is the gold standard marker protein for mitochondrial mass [30, 31].

Whether the reduction in mitochondrial mass is a consequence of lower mitochondrial biogenesis or higher mitophagy (or both mechanisms) remains to be elucidated. PGC-1*α* is a master regulator of mitochondrial biogenesis [32, 33]. Deletion of the PGC-1*α* gene causes spontaneous colitis and increases susceptibility to experimental colitis, and also PPAR-*γ*, the target of PGC-1*α*, was reported to be downregulated in UC [34]. Nitric oxide (NO), a well-known effector molecule with diverse functions, has been reported to be a stimulator of mitochondrial biogenesis [35]. NO has also been associated with the initiation and maintenance of inflammation in human IBD [36].

The second principal mechanism to explain the reduction in mitochondrial mass is an increase in autophagy or more specifically mitophagy, a process which removes damaged mitochondria. Indeed, a plethora of proteins associated with autophagy contribute to the pathogenesis of IBD [37–39]. We found normal amounts of the UQCRC2 subunit of complex III in our patient cohort. This might reflect compensatory upregulation in response to the reduced mitochondrial mass present in IBD. Notably, it was very recently proposed that complex III is a tuner of autophagy [40]. Usually, mitochondrial dysfunction causes a reduction of autophagy because autophagy is an ATP-consuming process. It would be very interesting if high complex III levels are a signal for the removal of damaged mitochondria. Antimycin A and myxothiazol reduce autophagy via complex III. Inhibition of the other OXPHOS complexes had no effect on autophagy. ATG16L1 is a component of a large protein complex essential for autophagy. In genome-wide association studies, variants in ATG16L1 have been linked to IBD (OMIM#610767) [41, 42]. ATG16L1 regulates mitochondrial antiviral signaling- (MAVS-) dependent type I interferon (IFN-I) production [43]. ATG16L1 deficiency causes mitochondrial defects in human macrophages. Furthermore, the authors reported a reduced number of mitochondria in ATP16L1-deficient cells [44].

Complex I and complex II subunits were more severely reduced than those of the other OXPHOS complexes and VDAC1 in both CD and UC patients. Therefore, an additional dysfunction causing this more pronounced complex I and II reduction might be present in IBD. Complex II is exclusively encoded by the nuclear DNA. Therefore, the combined reduction of complex I and II indicates the multifactorial cause of IBD. It is also known that complex I and complex II do not form respiratory chain supercomplexes together [45]. Our data are in line with those of Haberman et al., who demonstrated reduced complex I expression in UC [24]. In contrast, Sifroni et al. reported diminished complex II, III, and IV in UC patients but normal complex I activity [18]. Complex I is the largest OXPHOS complex, consisting of 44 different subunits encoded by both mitochondrial and nuclear DNA [46]. Therefore, it was previously proposed that complex I is the complex most prone to damage, simply because of its size. In addition, complex I is a multifunctional protein involved in several pathways fundamental for IBD pathogenesis, such as apoptosis. Caspase 3 cleaves the NDUFS1 subunit of complex I to induce apoptosis [47, 48]. In a UC model, granzyme A was increased prior to macroscopic disease manifestation [49]. Granzyme A is another protein able to cleave complex I and induce apoptosis [50]. Stamp et al. showed that a reduction in complex I is associated with an increased rate of stem cell cycle reentry in the mouse colon, suggesting that these changes in stem cell homeostasis could have an impact on age-associated pathologies of the colon [51]. Methylation-controlled J protein (MCJ) acts as a natural inhibitor of complex I. Loss of MCJ results in aggravated disease with a change in microbiota composition and altered intestinal permeability, suggesting that MCJ plays a protective function during intestinal inflammation [52]. Importantly, both of the main entry points for electrons, complex I and complex II (FADH_2_), are downregulated in IBD, indicating that OXPHOS is indeed reduced in IBD and not compensated via complex II. Santhanam et al. stated that a reduction in complex II activity appears to be a specific change in UC [17]. In a recent study, a marked decrease in MT-CO1 staining in the supranuclear region of the superficial epithelium relative to the subnuclear region was reported in 56% of patients with CD and 60% patients with UC [53]. However, the study did not analyze subunits of the other OXPHOS complexes. The authors further found that mitochondrial dysfunction alters intestinal epithelial metabolism of hepatic acylcarnitine species. NDUFAB1, also termed acyl carrier protein, is a subunit of complex I. Furthermore, NDUFAB1 is involved in Fe-S cluster biogenesis and protein lipoylation [54]. Therefore, it is possible in principle that low levels of NDUFAB1/complex I might influence complex II, which carries a [2Fe-2S] and a [4Fe-4S] cluster [55, 56].

In general, a trend to higher levels of OXPHOS complex subunits was present in adult patients with UC (rectum; ascending colon) and CD (terminal ileum; ascending colon) compared to pediatric patients, whereas VDAC1 showed a trend to lower amounts in adults. In adults, complex I was reduced in the rectum of UC patients and complex IV in the ascending colon of CD patients.

We hypothesize that an altered inner/outer membrane ratio might at least be partially responsible for the observed age-dependent phenotype. Altered mitochondrial (cristae) morphology as well as changes in proteins involved in fission/fusion was reported for IBD. A reduction of all OXPHOS complexes can be a consequence of reduced inner membrane folding [57–59]. MFN1, MFN2, OPA1, and p-DRP1 levels increased in a model of DSS-induced colitis [60, 61]. A mitochondrial fission inhibitor ameliorated DSS- and DNBS-induced murine colitis [62]. Whether a therapeutic intervention that shifts mitochondrial turnover to either fission or fusion would be beneficial remains to be elucidated. Interestingly, it was proposed that mitochondrial fusion of healthy and damaged mitochondria helps to maintain a functional mitochondrial compartment [63].

Studies have shown that the mitochondrial network of aged animals is often more heterogeneous, fragmented, and comprised of large, swollen mitochondria that cannot be eliminated by mitophagy [63]. We hypothesize that mitochondrial swelling might be present in IBD of adult patients. Swelling would cause a decrease in VDAC1 because the outer membrane fraction would decline. However, it was also reported that fragmentation and swelling induce a loss of OXPHOS complexes, which is in contrast to our findings.

By stratifying the expression levels according to severity of inflammation, lower expression levels in IBD patients were seen, but more severe inflammation did not correlate with a reduction of mitochondrial mass and/or expression level of the OXPHOS complexes. Patients with the highest HSS values exhibited higher expression levels than those with only moderate degrees of inflammation. A possible mechanism could be that the mitochondrial mass increases as a compensatory mechanism due to dysfunctionality within the respiratory chain.

Another possible explanation is that ROS, produced by the respiratory chain, activate the NLRP3 inflammasome, which is a known activator of excessive inflammatory responses. Under physiologic conditions there is only a low leakage of ROS, but damage to the respiratory chain produces higher amounts of ROS and stronger activation of the NLRP3 inflammasome [64]. Since it is not known, if there is an increased production of ROS within the given samples, this is only speculative.

The inflammation by using the HSS score in adults was not more severe than in pediatric patients. The HSS score was 2 (mild-moderate inflammation) in most of the cases and balanced between adults and children (data not shown). However, due to the low case numbers in the higher HSS classes and the heterogeneous results in the subgroups, the results should be interpreted carefully.

Limitations of our study include the lack of enzyme measurements. We cannot exclude defects in the activity of respiratory chain enzymes. We employed IHC staining since it enables reliable analysis of OXPHOS respiratory chain defects at the level of the single cell, thus providing a major advantage over enzymatic measurements, which do not allow discrimination between different cell types [27, 65, 66]. In addition, we cannot provide clinical data, including fecal calprotectin measurements. However, an HSS methodology was chosen, as this is the gold standard for evaluating the degree of inflammation. Low case numbers in the higher HSS classes only allowed for descriptive analysis. However, to our knowledge, this is the most comprehensive study evaluating all complexes and mitochondrial mass in pediatric and adult IBD.

## 5. Conclusion

Reduced mitochondrial mass is present in UC and CD compared to controls. This is potentially a result of alterations of mitochondrial biogenesis or mitophagy. Reductions were more pronounced in older patients compared to pediatric patients and more prominent in UC than CD. Complex I and II are more severely compromised than the other OXPHOS complexes. This has potential therapeutic implications, since treatments boosting biogenesis or influencing mitophagy could be beneficial for IBD treatment. Additionally, substances specifically stimulating complex I activity should be tested in IBD treatment.

## Figures and Tables

**Table 1:** Patient characteristics.

Group	Number of patients	Age, mean (range) (years)	Sex, female (%)
p-IBD	28 (CD = 15, CU = 13)	12.6 (3-17)	16 (57)
a-IBD	39 (CD = 19, CU = 20)	52.7 (27-89)	22 (51)
Pediatric controls	18	12.5 (3-17)	10 (59)
Adult controls	39	53.6 (20-82)	26 (65)
Total	124		

p-IBD: children and adolescents with inflammatory bowel disease (IBD); a-IBD: adults with IBD; pediatric controls and adult controls; CD: Crohn's disease; UC: ulcerative colitis.

**Table 2:** Expression levels of OXPHOS complexes I to V and VDAC1 (porin) in the terminal ileum in Crohn's disease (CD).

	CI	CII	CIII	CIV	CV	VDAC1
Expression level, mean (SD)						
CD	138 (68)	115 (63)	166 (61)	206 (50)	168 (68)	139 (44)
Controls	175 (65)	156 (62)	177 (48)	233 (44)	178 (51)	150 (43)
Reduction controls- CD	37 (21%)	41 (26%)	11 (6%)	27 (12%)	10 (6%)	11 (7%)
*p* value	0.053	0.030^∗^	0.895	0.081	0.850	0.442

It is shown as mean with standard deviation (SD) and reduction of expression level in CD patients compared to the control group. ^∗^*p* < 0.05.

**Table 3:** Expression levels of OXPHOS complexes I to V and VDAC 1 (porin) in the rectum in ulcerative colitis (UC).

	CI	CII	CIII	CIV	CV	VDAC1
Expression level, mean (SD)						
UC	114 (69)	110 (70)	166 (65)	202 (42)	151 (54)	113 (48)
Controls	200 (75)	151 (73)	192 (67)	229 (65)	180 (54)	149 (50)
Reduction controls- UC	86 (43%)	41 (27%)	26 (14%)	27 (12%)	29 (16%)	36 (24%)
*p* value	<0.001^∗^	0.024^∗^	0.182	0.013^∗^	0.182	0.009^∗^

It is shown as mean with standard deviation (SD) and reduction of expression level in CD patients compared to control group. ^∗^*p* < 0.05.

**Table 4:** Expression levels of OXPHOS complexes I to V and VDAC1 (porin) in Crohn's disease (CD), ulcerative colitis (UC), and controls in the ascending colon.

	CI	CII	CIII	CIV	CV	VDAC1
Expression level, mean (SD)						
CD (*n* = 34)	126 (69)	100 (65)	155 (66)	212 (48)	152 (72)	143 (45)
UC (*n* = 33)	130 (80)	105 (66)	150 (58)	195 (58)	143 (62)	113 (48)
Control (*n* = 57)	195 (73)	160 (75)	181 (59)	227 (54)	192 (60)	153 (42)
Reduction controls- IBD	67 (34%)	58 (36%)	29 (16%)	23 (10%)	45 (23%)	24 (16%)
*p* value	<.001^∗^	0.002^∗^	0.207	0.079	0.020^∗^	0.005^∗^

It is shown as mean with standard deviation (SD) and reduction of expression level in IBD patients (CD and UC) compared to the control group. ^∗^*p* < 0.05.

## Data Availability

The data used to support the findings of this study are included within the article.

## References

[1] Conrad K., Roggenbuck D., Laass M. W. (2014). Diagnosis and classification of ulcerative colitis. *Autoimmunity Reviews*.

[2] Khor B., Gardet A., Xavier R. J. (2011). Genetics and pathogenesis of inflammatory bowel disease. *Nature*.

[3] Uniken Venema W. T., Voskuil M. D., Dijkstra G., Weersma R. K., Festen E. A. (2017). The genetic background of inflammatory bowel disease: from correlation to causality. *The Journal of Pathology*.

[4] Baumgart D. C., Carding S. R. (2007). Inflammatory bowel disease: cause and immunobiology. *Lancet*.

[5] Gunther C., Josenhans C., Wehkamp J. (2016). Crosstalk between microbiota, pathogens and the innate immune responses. *International Journal of Medical Microbiology*.

[6] Maloy K. J., Powrie F. (2011). Intestinal homeostasis and its breakdown in inflammatory bowel disease. *Nature*.

[7] Sauer C. G., Kugathasan S. (2010). Pediatric inflammatory bowel disease: highlighting pediatric differences in IBD. *The Medical Clinics of North America*.

[8] Aloi M., Lionetti P., Barabino A. (2014). Phenotype and disease course of early-onset pediatric inflammatory bowel disease. *Inflammatory Bowel Diseases*.

[9] van Limbergen J., Russell R. K., Drummond H. E. (2008). Definition of phenotypic characteristics of childhood-onset inflammatory bowel disease. *Gastroenterology*.

[10] McBride H. M., Neuspiel M., Wasiak S. (2006). Mitochondria: more than just a powerhouse. *Current Biology*.

[11] Beltrán B., Nos P., Dasí F. (2010). Mitochondrial dysfunction, persistent oxidative damage, and catalase inhibition in immune cells of naïve and treated Crohnʼs disease. *Inflammatory Bowel Diseases*.

[12] Pavlick K. P., Laroux F. S., Fuseler J. (2002). Role of reactive metabolites of oxygen and nitrogen in inflammatory bowel disease1, 2. *Free Radical Biology & Medicine*.

[13] Wang A., Keita Å. V., Phan V. (2014). Targeting mitochondria-derived reactive oxygen species to reduce epithelial barrier dysfunction and colitis. *The American Journal of Pathology*.

[14] Matondo A., Kim S. S. (2018). Targeted-mitochondria antioxidants therapeutic implications in inflammatory bowel disease. *Journal of Drug Targeting*.

[15] Lih-Brody L., Powell S. R., Collier K. P. (1996). Increased oxidative stress and decreased antioxidant defenses in mucosa of inflammatory bowel disease. *Digestive Diseases and Sciences*.

[16] Bär F., Bochmann W., Widok A. (2013). Mitochondrial gene polymorphisms that protect mice from colitis. *Gastroenterology*.

[17] Santhanam S., Rajamanickam S., Motamarry A. (2012). Mitochondrial electron transport chain complex dysfunction in the colonic mucosa in ulcerative colitis. *Inflammatory Bowel Diseases*.

[18] Sifroni K. G., Damiani C. R., Stoffel C. (2010). Mitochondrial respiratory chain in the colonic mucosal of patients with ulcerative colitis. *Molecular and Cellular Biochemistry*.

[19] Boyapati R. K., Dorward D. A., Tamborska A. (2018). Mitochondrial DNA is a pro-inflammatory damage-associated molecular pattern released during active IBD. *Inflammatory Bowel Diseases*.

[20] Ussakli C. H., Ebaee A., Binkley J. (2013). Mitochondria and tumor progression in ulcerative colitis. *Journal of the National Cancer Institute*.

[21] Restivo N. L., Srivastava M. D., Schafer I. A., Hoppel C. L. (2004). Mitochondrial dysfunction in a patient with Crohn disease: possible role in pathogenesis. *Journal of Pediatric Gastroenterology and Nutrition*.

[22] Vanderborght M., Nassogne M. C., Hermans D. (2004). Intractable ulcerative colitis of infancy in a child with mitochondrial respiratory chain disorder. *Journal of Pediatric Gastroenterology and Nutrition*.

[23] Mottawea W., Chiang C. K., Mühlbauer M. (2016). Altered intestinal microbiota-host mitochondria crosstalk in new onset Crohn's disease. *Nature Communications*.

[24] Haberman Y., Karns R., Dexheimer P. J. (2019). Ulcerative colitis mucosal transcriptomes reveal mitochondriopathy and personalized mechanisms underlying disease severity and treatment response. *Nature Communications*.

[25] Özsoy M., Zimmermann F. A., Feichtinger R. G. (2020). Changes in the expression of oxidative phosphorylation complexes in the aging intestinal mucosa. *Experimental Gerontology*.

[26] Zimmermann F. A., Neureiter D., Feichtinger R. G. (2016). Deficiency of respiratory chain complex I in Hashimoto thyroiditis. *Mitochondrion*.

[27] Zimmermann F. A., Mayr J. A., Neureiter D. (2009). Lack of complex I is associated with oncocytic thyroid tumours. *British Journal of Cancer*.

[28] Dobler D., Friedrich S., Pauly M. (2018). Nonparametric MANOVA in Mann-Whitney effects.

[29] Happ M., Zimmermann G., Brunner E., Bathke A. C. (2020). Pseudo-ranks: how to calculate them efficiently inR. *Journal of Statistical Software*.

[30] Alston C. L., Veling M. T., Heidler J. (2020). Pathogenic Bi-allelic Mutations in _NDUFAF8_ Cause Leigh Syndrome with an Isolated Complex I Deficiency. *American Journal of Human Genetics*.

[31] Ng Y. S., Thompson K., Loher D. (2020). Novel MT-ND gene variants causing adult-onset mitochondrial disease and isolated complex I deficiency. *Frontiers in Genetics*.

[32] Ho G. T., Aird R. E., Liu B. (2018). MDR1 deficiency impairs mitochondrial homeostasis and promotes intestinal inflammation. *Mucosal Immunology*.

[33] Scarpulla R. C. (2011). Metabolic control of mitochondrial biogenesis through the PGC-1 family regulatory network. *Biochimica et Biophysica Acta*.

[34] DOU X., XIAO J., JIN Z., ZHENG P. (2015). Peroxisome proliferator-activated receptor-*γ* is downregulated in ulcerative colitis and is involved in experimental colitis-associated neoplasia. *Oncology Letters*.

[35] Litvinova L., Atochin D. N., Fattakhov N., Vasilenko M., Zatolokin P., Kirienkova E. (2015). Nitric oxide and mitochondria in metabolic syndrome. *Frontiers in Physiology*.

[36] Kolios G., Valatas V., Ward S. G. (2004). Nitric oxide in inflammatory bowel disease: a universal messenger in an unsolved puzzle. *Immunology*.

[37] Lassen K. G., Xavier R. J. (2017). Genetic control of autophagy underlies pathogenesis of inflammatory bowel disease. *Mucosal Immunology*.

[38] Ma C., Storer C. E., Chandran U. (2021). Crohn's disease-associated ATG16L1 T300A genotype is associated with improved survival in gastric cancer. *eBioMedicine*.

[39] Shao B. Z., Yao Y., Zhai J. S., Zhu J. H., Li J. P., Wu K. (2021). The role of autophagy in inflammatory bowel disease. *Frontiers in Physiology*.

[40] Jin S. (2011). Mitochondrial complex III: tuner of autophagy. *Chemistry & Biology*.

[41] Lavoie S., Conway K. L., Lassen K. G. (2019). The Crohn's disease polymorphism, ATG16L1 T300A, alters the gut microbiota and enhances the local Th1/Th17 response. *eLife*.

[42] Salem M., Ammitzboell M., Nys K., Seidelin J. B., Nielsen O. H. (2015). ATG16L1: a multifunctional susceptibility factor in Crohn disease. *Autophagy*.

[43] Grimm W. A., Messer J. S., Murphy S. F. (2016). The Thr300Ala variant in ATG16L1 is associated with improved survival in human colorectal cancer and enhanced production of type I interferon. *Gut*.

[44] Shih D., Kanazawa Y., Hamill A., McGovern D., Fukata M., Targan S. (2016). P-189 ATG16L1 deficiency leads to mitochondria defect and increased oxidative state in mice and human macrophages. *Inflammatory Bowel Diseases*.

[45] Novack G. V., Galeano P., Castaño E. M., Morelli L. (2020). Mitochondrial Supercomplexes: physiological organization and dysregulation in age-related neurodegenerative disorders. *Front Endocrinol (Lausanne)*.

[46] Fiedorczuk K., Letts J. A., Degliesposti G., Kaszuba K., Skehel M., Sazanov L. A. (2016). Atomic structure of the entire mammalian mitochondrial complex I. *Nature*.

[47] Nunes T., Bernardazzi C., de Souza H. S. (2014). Cell death and inflammatory bowel diseases: apoptosis, necrosis, and autophagy in the intestinal epithelium. *BioMed Research International*.

[48] Ricci J. E., Muñoz-Pinedo C., Fitzgerald P. (2004). Disruption of mitochondrial function during apoptosis is mediated by caspase cleavage of the p75 subunit of complex I of the electron transport chain. *Cell*.

[49] HIRAYASU H., YOSHIKAWA Y., TSUZUKI S., FUSHIKI T. (2007). A role of a lymphocyte tryptase, granzyme A, in experimental ulcerative colitis. *Bioscience, Biotechnology, and Biochemistry*.

[50] Martinvalet D., Dykxhoorn D. M., Ferrini R., Lieberman J. (2008). Granzyme A Cleaves a Mitochondrial Complex I Protein to Initiate Caspase- Independent Cell Death. *Cell*.

[51] Stamp C., Whitehall J. C., Smith A. L. M. (2021). Age-associated mitochondrial complex I deficiency is linked to increased stem cell proliferation rates in the mouse colon. *Aging Cell*.

[52] Pascual-Itoiz M. A., Peña-Cearra A., Martín-Ruiz I. (2020). The mitochondrial negative regulator MCJ modulates the interplay between microbiota and the host during ulcerative colitis. *Scientific Reports*.

[53] Smith S. A., Ogawa S. A., Chau L. (2021). Mitochondrial dysfunction in inflammatory bowel disease alters intestinal epithelial metabolism of hepatic acylcarnitines. *The Journal of Clinical Investigation*.

[54] Masud A. J., Kastaniotis A. J., Rahman M. T., Autio K. J., Hiltunen J. K. (2019). Mitochondrial acyl carrier protein (ACP) at the interface of metabolic state sensing and mitochondrial function. *Biochimica et Biophysica Acta, Molecular Cell Research*.

[55] Albracht S. P. (1980). The prosthetic groups in succinate dehydrogenase Number and stoichiometry. *Biochimica et biophysica acta Reviews on cancer*.

[56] Na U., Yu W., Cox J. (2014). The LYR factors SDHAF1 and SDHAF3 mediate maturation of the iron-sulfur subunit of succinate dehydrogenase. *Cell Metabolism*.

[57] Bou-Fersen A. M., Anim J. T., Khan I. (2008). Experimental colitis is associated with ultrastructural changes in inflamed and uninflamed regions of the gastrointestinal tract. *Medical Principles and Practice*.

[58] Modica-Napolitano J. S., Steele G. D., Chen L. B. (1989). Aberrant mitochondria in two human colon carcinoma cell lines. *Cancer Research*.

[59] Wilson P. D., Franks L. M. (1975). The effect of age on mitochondrial ultrastructure and enzymes. *Advances in Experimental Medicine and Biology*.

[60] Cipolat S., de Brito O. M., Dal Zilio B., Scorrano L. (2004). OPA1 requires mitofusin 1 to promote mitochondrial fusion. *Proceedings of the National Academy of Sciences of the United States of America*.

[61] Mancini N. L., Goudie L., Xu W. (2020). Perturbed mitochondrial dynamics is a novel feature of colitis that can be targeted to lessen disease. *Cellular and Molecular Gastroenterology and Hepatology*.

[62] Goudie L. J. L., Mancini N., Blote K. R., Wang A., McKay D. M., Shearer J. (2018). A novel mitochondrial fission inhibitor ameliorates DSS and DNBS Induced Murine Colitis. *Induced Murine Colitis.*.

[63] Sharma A., Smith H. J., Yao P., Mair W. B. (2019). Causal roles of mitochondrial dynamics in longevity and healthy aging. *EMBO Reports*.

[64] Zhang T., Ding S., Wang R. (2021). Research progress of mitochondrial mechanism in NLRP3 inflammasome activation and exercise regulation of NLRP3 Inflammasome. *International Journal of Molecular Sciences*.

[65] Feichtinger R. G., Neureiter D., Skaria T. (2017). Oxidative phosphorylation system in gastric carcinomas and gastritis. *Oxidative Medicine and Cellular Longevity*.

[66] Feichtinger R. G., Zimmermann F. A., Mayr J. A. (2011). Alterations of respiratory chain complexes in sporadic pheochromocytoma. *Frontiers in Bioscience (Elite Edition)*.

[67] Schneider A. (2021). Expression of oxidative phosphorylation complexes and mitochondrial mass in pediatric and adult infammatory bowel disease- abstract. *Monatsschrift Kinderheilkunde*.

